# Prevalence and Characteristics of Orthorectic Disorders in Adolescence and Young People: Polish Preliminary Studies

**DOI:** 10.3390/nu13051568

**Published:** 2021-05-07

**Authors:** Natalia Kaźmierczak-Wojtaś, Rafał Patryn, Anna Zagaja, Mariola Drozd, Antoni Niedzielski

**Affiliations:** 1 Department of Psychology, Medical University of Lublin, 20-059 Lublin, Poland; nataliakazmierczakwojtas@umlub.pl (N.K.-W.); antoniniedzielski@umlub.pl (A.N.); 2 Department of Humanities and Social Medicine, Medical University of Lublin, 20-059 Lublin, Poland; rafalpatryn@umlub.pl (R.P.); annazagaja@umlub.pl (A.Z.)

**Keywords:** risk of orthorectic behaviors, adolescent, young people, health

## Abstract

The aim of this work was to assess orthorectic behaviors among young people and to evaluate their attitudes towards caring for their health. The study was conducted in 2019 on a group of 538 respondents aged 16–35. After analysis, 65 questionnaires were eliminated from further research, and the assessment of orthorectic disorders was performed using a method based on the modified ORTO-15 questionnaire on a group of 473 respondents. A large percentage of them exhibited an increased risk of orthorectic behaviors (32.8), which was higher among women than men (34.7% and 28.2%, respectively). People with higher risks of orthorectic disorders significantly more often reduced their consumption of foods high in fats and sugars. Attitudes of people with orthorectic disorders towards health care proved neutral, with a tendency to be positive. Nutritional behaviors observed in the studied group show some irregularities, which indicates the need for preventive and educational measures aimed at increasing awareness of the role of proper nutrition among young people. The obtained results may be the basis for further research on ON symptoms. One of the major areas of future research would be to create a reliable diagnostic tool which would allow for distinguishing between orthorexia and overdiagnosis.

## 1. Introduction

Mental orthorexia (*Orthorexia nervosa*—ON, from Greek *ortho*—correct, proper; *orexis*—appetite, hunger) is defined as a pathological fixation (obsession; care; preoccupation) on eating proper and healthy food. The obsession with healthy eating results from the desire to improve one’s health and maintain their well-being.

The term was first introduced in 1997 by an American physician, Dr. Steven Bratman, who described it as a “fixation on eating healthy foods” [1]. It is worth noting that Bratman did not base the description of ON on empirical research, but on personal experience. In his book titled “Health Food Junkies”, he presents his struggles with ON along with cases of other people whose strict diets led them to this disorder. In the book, he describes the symptoms that characterize this disorder, including an obsessive focus on the quality of consumed products and the ways in which meals are prepared [1]. Additional symptoms of the orthorectic disorder include thoroughly analyzing the source, production and packaging process of foods available on the market [2,3] and excluding foods considered unhealthy or impure [4,5,6,7]. People with orthorectic disorders often limit or completely eliminate specific food groups, such as meat, dairy products, grains, ready meals, non-seasonal products [1], genetically modified foods high in fat, salt or sugars, foods that contain pesticides and sensitizing substances [8]. It is also common practice to eat food derived exclusively from organic farming that does not contain artificial substances or preservatives [2,9]. For people with an ON disorder, food is primarily a source of health, not pleasure. The concept of “healthy” food is an individual matter and can change depending on a person’s mood, beliefs or knowledge about food and nutrition. For this reason, it is difficult to clearly define what makes a given product perceived as “healthy”.

The development of ON may be triggered by a doctor’s/dietitian’s recommendations regarding the use of a proper diet, the desire to overcome chronic diseases, negative life events and mental disorders. Risk factors can include the use of alternative diets (e.g., vegetarian, vegan, macrobiotic), nutritional restrictions or a desire to live in harmony with nature [1,2]. From a medical point of view, a direct effect of a selective approach to diet may cause nutrition deficiency, leading to malnutrition and weight loss [1,2,10,11,12]. In extreme cases, ON can have the same consequences as severe anorexia, i.e., osteopenia, anemia, hyponatremia, hypokalemia, metabolic acidosis, testosterone deficiency, bradycardia, pancytopenia, subcutaneous emphysema and pneumothorax [1,10,11]. Bratman also presents numerous mental health-related consequences. Additionally, he writes about food as a religion, where choosing food products and food intake can be performed at a spiritual level and the deviation from an ideal diet may be accompanied by a feeling of failure [1].

Orthorexia does not have an official definition, it has not been included in the current International Statistical Classification of Diseases and Related Health Problems or the Diagnostic and Statistical Manual of Mental Disorders (ICD-10, DSM-5), and the diagnostic criteria cited in the subject’s literature are still only a proposal [5]. Many questions arise as to the relationship between ON and other nosological units. In the subject’s literature, ON is sometimes presented as an eating disorder (ED), sometimes as an obsessive-compulsive disorder (OCD) [13,14,15]. Scientists also suggest that the American Psychological Association (APA) should modify the eating disorder not otherwise specified (EDNOS) category and define new types of eating disorders [16]. There is also disagreement among researchers as to whether ON should be considered a separate disorder, a variant of a currently recognized disorder (simply) a disturbed eating behavior or a disorder at all [17,18]. Nevertheless, the National Eating Disorders Association (NEDA) has published official information on ON, emphasizing the need for further research in this area [19].

The available literature provides ambiguous empirical data regarding the prevalence of ON. It varies between countries and between different social groups within a given country. Research to date indicates that the tendency towards orthorectic behaviors in the general population ranges from 1% [20] to 75.2% [21]. On the other hand, research conducted among youth and students indicates ON prevalence ranging from 2.5% among German students [22] to over 88% among Brazilian dietitian students [23]. Among Polish students, the incidence of ON ranges from 52.6% [24] to 76.7% [25] using the original version of the ORTO-15 questionnaire and a threshold of 40 points. However, after lowering the threshold to 35, the incidence of orthorectic behavior decreased and ranged from 13.7% [24,26] to 28.3% [27,28]. The risk of the occurrence of orthorectic disorders measured by the Polish version of the ORTO-15 questionnaire reached 65.1% [29].

The aim of this study was to assess the prevalence of orthorectic behaviors in young people and to assess their attitudes towards caring for their health.

The following research variables were distinguished within the research model:

1. Orthorexia—a variable describing the level of the respondents’ obsessive thoughts about the quality of consumed foods, selective eating of chosen products and methods of preparing meals. To measure this variable, the ORTO-15 questionnaire was used.
2. Self-assessment of the presence of eating-related problems—a variable describing the respondents’ individual attitudes towards food. To measure this variable, the EAT-26 questionnaire was used. This variable is complex in nature and has three components: dieting, bulimia and food preoccupation, and oral control.
3. Concern for health—a variable describing the interest in one’s own health and the health value of food. Here, to measure this variable, the Health Concern Scale was used.

## 2. Material and Methods

### 2.1. The Following Study Was Conducted between January and April 2019 by a Licensed Psychologist

The research was carried out among young people (pupils, students, employees and students who were also employed) aged 16–35 (*n* = 538) inhabiting the Lubelskie Voivodeship. The study was conducted in 3 secondary schools (2 high schools, 1 technical high school; 5 grades altogether) and 3 universities (Medical University of Lublin, Catholic University of Lublin, the University of Economics and Innovation in Lublin). Schools and universities were selected at random, after taking into account all entities located in the Lubelskie Voivodeship. Afterwards, at each school, classes and groups of students were also randomly selected. To ensure a high return of completed questionnaires, the researchers selected classes and groups of students that could be reached directly (during classes). Teachers and other staff members aged up to 35 and present on the day of the study were also asked to participate. In total, the questionnaire was distributed to 600 respondents; however, only 538 of them returned it, setting the participation rate at 89.7%. After initial verification, 473 complete and correctly filled out questionnaires were qualified for further analysis.

Persons under 16 years of age were excluded from the study (*n* = 7) due to the insufficient impact on dietary choices [4]. The reason that we concentrated on late adolescence (16–18 year) and early adulthood (18–35 year) is because the risk of orthorectic disorders, as was indicated by numerous authors [4,16,30,31], most frequently occurs both in young and slightly older people. Additionally, from a psychological point of view, this period may prove crucial because young adults are becoming more independent from their parents and beginning to make their own food choices. People who obtained a positive result in the Eating Attitudes Test (EAT-26) (i.e., 20 points or more; *n* = 47) were also excluded from further analyses, because they are at risk of developing eating disorders, and this could have altered the study’s results.

This criterion was also applied in other Polish studies [29]. Thanks to this, the authors could elicit respondents whose eating behavior was not disturbed. Questionnaires that were considered non-diagnostic (*n* = 11) were also excluded from further analysis (e.g., those incomplete or filled out in an unreliable manner).

### 2.2. The Study Was Conducted Using the Following Research Tools

The only tool for determining the risk of orthorexic disorders at the time of the study was the Bratman test, which is not a diagnostic tool; the EHQ questionnaire, which, at that time, lacked a Polish language version; and the ORTO-15 questionnaire in the Polish language version.

#### 2.2.1. Questionnaire ORTO-15

The ORTO-15 questionnaire [3] consists of 15 items describing the severity of orthorectic behaviors. It includes cognitive, emotional and clinical aspects of orthorectic disorders. Respondents provide answers using a 4-point Likert scale—always, often, sometimes, never—to which point values from 1 to 4 are assigned. The authors determined a threshold value of 40 points. A lower score indicates a tendency towards orthorectic disorders.

The ORTO-15 questionnaire was adapted to Polish conditions by Brytek-Matera et al. [29]. As a result of analyses, the authors received a 9-item tool, whose Cronbach’s alpha value was 0.644. The obtained result indicates acceptable reliability but does not meet the Nunnally criterion (acceptable level of reliability threshold α > 0.70) [32]. In connection with the above, it was decided to re-check the reliability of the tool in question and, on the basis of the obtained results, remove certain items.

Because the calculated Cronbach alpha reliability coefficient for the original 15-item version of the ORTO-15 questionnaire was 0.259, and 0.651 for the Polish version, a decision was made to remove items from the questionnaire that were the least correlated with the overall result. Finally, a 6-item tool (items 4, 6, 10, 11, 12, 14) was obtained (ORTO-6), where the reliability increased to 0.696. This is a satisfactory result and close to the acceptable value of 0.7 required for scientific research [33]. In this way, the ORTO-6 questionnaire was created, which included 4 ranges (lowest possible score of 6 points).

- 6–7 points—orthorectic disorders;
- 8–11points—tendency towards orthorexy;
- 12–15 points—appropriate eating habits;
- 16–24 points—low food interest.

Recently, a new 6-item (item 3, 4, 7, 10, 11, 12) modification of the ORTO-15 questionnaire became available, called ORTO-R [34].

The study used the authors’ own modification of the ORTO-15 questionnaire, the ORTO-6, which comprises the following questions:

Are your eating choices conditioned by your worry about your health status?
Are you willing to spend more money to have healthier food?
Do you think that the conviction to eat only healthy food increases self-esteem?
Do you think that eating healthy food changes your lifestyle (frequency of eating out, friends, …)?
Do you think that consuming healthy food may improve your appearance?
Do you think that on the market there is also unhealthy food?

#### 2.2.2. Eating Attitudes Test-26 (EAT-26)

EAT-26 is a self-assessment test, most often used as a screening tool to detect eating disorder symptoms in the general population. It consists of 26 items and 3 subscales: dieting, bulimia and food preoccupation and oral control. Respondents provide answers on a 6-point scale, from “always” to “never”. The maximum number of points for each item is “3”, which is synonymous with the strongest test feature expressed. The final result is the sum of all subscales. A score equal to or above 20 points suggests the risk of disturbances in the fulfillment of food needs and attitudes towards one’s own body. Alpha Cronbach’s reliability for the entire scale is 0.84 [35].

#### 2.2.3. Health Concern Scale (HCS)

HCS [36] consists of 10 statements describing interest in health and the relationship of excessive consumption of certain food products, e.g., sugar, fat, salt or food additives, with certain diseases, e.g., hypertension and ischemic heart disease. Test subjects have a 7-point response scale, ranging from “definitely not” (1 point) to “definitely yes” (7 points). The item which contained negation (9) was recoded. The results were calculated for the entire HCS scale. The more points a respondent received, the greater were his/her health concerns. Based on the sum of points from the HCS scale, people with negative, neutral or positive health attitudes were distinguished. As the division criterion, the authors established ⅓ and ⅔ of the points range (negative attitude—10–29, neutral attitude 30–50 and positive attitude 51–70 points).

Obtained results were subjected to statistical analysis using the IBM SPSS Statistics version 23. For analysis, the authors applied the ANOVA test, Tukeya test and Cronbach’s alpha.

## 3. Results

### 3.1. Division of the Study Group Based on the ORTO-6 Questionnaire

The study group was divided based on statistical and empirical criteria. According to the statistical criterion established by Kelley [37], the division of the research group was based on extremes (in order to assess the differences between them) so that at each of the extremities of the distribution of results (so-called “tails”) was 25–27% of the total number of cases. In such a situation, the power level of performed tests is the highest. An additional empirical criterion determined after the authors of the Polish validation of the ORTO-15 questionnaire [24], which referred to the clinical picture, was also added. According to the authors, the best correlations in recognizing the risk of orthorectic disorders and the risk of eating disorders are obtained when the cut-off threshold is set at 35 points. This leads to the separation of a group of people with orthorectic disorders consisting of 5% of the respondents. Accordingly, in the first extreme group—“tendency to orthorectic disorders”—an additional empirical criterion of 5% was used, thanks to which the “with the dominance of the orthorectic symptoms” group was distinguished.

Applying the above criteria, four subgroups were identified among the respondents: those with the dominance of orthorectic symptoms (3.6%), people with a tendency towards orthorectic disorders (29.2%), people who eat properly (44.4%) and those with low food interest (22.8%).

The vast majority of the respondents were female (70%). The majority of the respondents, both male and female, had proper eating habits (47.4% and 37.3%, respectively). The dominance of orthorectic symptoms was twice as likely to appear among women (4.2%) than among men (2.1%). It is worth mentioning that the tendency towards orthorectic disorders does not exhibit such differences between women (30.5%) and men (26.1%). What is interesting is that the occurrence of low food interest is 1.93 times higher than among women. Detailed data are presented in Table 1.

### 3.2. Dietary Restrictions

Most frequently, respondents limited the general amount of the consumed food (67.2%), sugar and sweets (65.3%), food containing high amounts of fat (60%) and fat (57.7%). Far fewer respondents indicated other products.

Afterwards, a statistical analysis was performed to determine the relationship between people showing orthorectic tendencies and dietary restrictions.

The Chi-2 test showed a statistically significant correlation between the group in which orthorectic symptoms are dominant and limiting foods with significant amounts of fat (Chi-2 = 56.331; *p* = 0.000), fats (Chi-2 = 33.465; *p* = 0.000), sugar and sweets (Chi-2 = 35.311; *p* = 0.000), meat and sausages (Chi-2 = 15.118; *p* = 0.002) and the total amount of food consumed (Chi-2 = 8.255; *p* = 0.041). This indicates that there are statistically significant differences between those at risk of orthorectic disorders and dietary restrictions. In the case of other dietary products, results proved statistically insignificant.

Detailed data are presented in Table 2.

Foods containing high amounts of fat and general fat consumption are significantly more limited by people with orthorexia than by other respondents (88.2% and 82.4%, respectively). Similarly, regarding the consumption of sugar and sweets, those with orthorexia (82.4%) and a tendency towards orthorexia (81.9%) tended to limit sweets more compared to other study participants. Analogous to this, albeit to a lesser extent, is the situation pertaining to meat and sausage consumption. A statistically significant correlation was observed between respondents in whom orthorectic symptoms were dominant and limiting the total amount of food consumed—limiting total food intake was observed more frequently among respondents with a tendency (74.6%) towards orthorexia than those not focused on food (57.4%). Detailed data are presented in Table 3.

### 3.3. Caring for Health

The overall score for caring for health was calculated using the Health Concern Scale (HCS). The respondents achieved results in the range of 10 to 70. The average result was 45 (SD = 12.01). The higher the average value presented by the HCS, the more positive attitudes are presented by the respondents. Obtained data indicate that respondents mostly have neutral attitudes towards caring for their health. Detailed data are presented in Table 4.

Variance analysis revealed that respondents with a tendency towards orthorexia obtained significantly higher scores on the HCS scale than people with low food interest. No statistically significant, linear correlation was observed between the overall result for ORTO and the Health Concern Scale (R = 0.084; *p* = 0.068).

## 4. Discussion

Orthorectic disorders are attracting more and more interest among representatives of various disciplines. Despite ongoing research and the increasing number of publications, it still remains a relatively poorly understood disorder. This is partly due to research conducted on non-clinical trials, a relatively small number of case studies [2,11,38,39] and also due the lack of a reliable diagnostic tool.

Despite the fact that the ORTO-15 questionnaire has recently met with criticism [40], a literature review proves that it is the most frequently applied tool to assess ON. Moreover, it should be noted that the lack of Polish language versions of other available methods (the use of the Bratman Test for scientific research is not recommended by the authors themselves, and the Polish version of the Eating Habits Questionnaire (EHQ) [41] did not exist at the time of conducting this study) left no choice and there was limited research on the use of the ORTO-15 tool. It can be assumed that other researchers also encounter similar problems.

The prevalence rate of orthorectic disorders among young people ranges from 2.5% among German students [22] to over 88% among Brazilian dietitian students [23] compared to the incidence of traditional eating disorders, whose prevalence is estimated at 0.4% for mental anorexia and 1–1.5% for bulimia nervosa (by the American Psychiatric Association in 2013), seems very high. However, our own research revealed that the incidence of orthorectic disorders is estimated at 3.6%, which indicates that it is not common. Similar results can be observed in studies conducted by other authors.

In the group of German students, the prevalence of orthorectic behavior ranges from 2.5% [22] to 3.3% [42]. Similar results were also reported by Oberle et al. [43] and Chard et al. [44] among American students (4.5% and 8%, respectively), and among Chinese students by He et al. [45]. However, due to the use of different measurement tools or different versions of the ORTO-15 questionnaire and, thus, different cut-off points, caution should be exercised in drawing conclusions from comparative analysis. Moreover, reported discrepancies may also result from cultural differences between research participants [16,46] as well as the heterogeneity of selected study groups (e.g., in resident doctors, the prevalence of ON is 45.5% [47], Ashtanga yoga practitioners—86% [48], Registered Dietitian Nutritionists (RDNs)—49.5% [49], general population—75.2% [21], students—75% [27]).

The problem with estimating the occurrence of orthorectic behaviors may be not only the use of an excessively high cut-off threshold in the ORTO-15 questionnaire, but also the fact that only two basic groups are distinguished on the basis of the obtained result, i.e., those exhibiting ON behaviors and those not showing the features of this disorder. Consequently, this translates into a high prevalence rate of ON in the study groups.

Therefore, the authors of this study decided to distinguish four groups based on the result obtained using the ORTO-6 questionnaire, i.e., orthorectic disorders, tendency towards orthorexia, proper nutrition and low food interest. This approach allows us not only to significantly reduce the level of prevalence of this disorder, but also to distinguish an increased risk group (i.e., tendency for orthorexia), to which preventive measures can be applied.

Additionally, this approach is also consistent with the latest position of Bratman [50], who distinguished two stages of ON, the so-called “healthy orthorexia”, when an individual is interested in healthy eating but does not show pathological features (which, in our own research, corresponds to the category “orthorexia tendency”) and “orthorexia nervosa”, when the individual is obsessed with pursuing a healthy diet (which corresponds to the “orthorexia” category). This solution was also used by F. Barthels, F. Meyer and R. Pietrowsky [51] in the Düsseldorf Orthorexia Scale (DOS). As a result, the prevalence rate of ON measured by the DOS scale ranges from 2.5% [22] to 10.5% [52]. Moreover, the early introduction of adequate measures among people with ON tendencies is especially important because orthorectic disorders may cause numerous negative physical and psychosocial consequences.

The authors’ modification of the ORTO-15, the ORTO-6 questionnaire, may help to solve current problems with measuring the level of orthorectic disorders.

According to our own research, orthorectic disorders occur more often among women than among men (4.2%, 2.1%, respectively), which is also confirmed in the research of other authors [53,54,55,56]. It should be noted, however, that men in previous orthorectic disorder studies usually comprised a smaller group. Therefore, these results may be less reliable for assessing gender differences.

The analysis of applied restrictions in food consumption carried out in this study allowed us to observe significant differences between distinguished groups in the type of limited/restricted food products. The most limited food products included products high in fats, sugar and sweets, meat and sausages and the total amount of food consumed. These products were definitely more often restricted by people with orthorectic disorders and/or a tendency towards orthorexia. Restrictions of meat and meat product consumption are confirmed by a relatively high content of substances that have an adverse effect on health, such as salts, additives, saturated fatty acids and fats, which are a source of cholesterol. High consumption of animal fat is one of the main causes of cardiovascular diseases, diabetes and obesity; therefore, its consumption during the day should be as low as possible [57]. Specialists from the International Agency for Research on Cancer (IARC) [58] also emphasize that greater than recommended meat consumption and the consumption of processed meat products can contribute to some cancers. According to the Polish Public Opinion Center (CBOS) report from [59], 34% of Poles limit the consumption of fatty foods, and 12% of meat. These results are similar to the results obtained in our own research in the non-food focus group (31.5% and 13.9% for fatty foods and meat, respectively). Similarly, a diet high in sugars, especially simple ones, can cause many adverse health effects. Excessive sugar and sweets consumption, due to the high calorific value, and often also the presence of fat, may cause overweight and obesity and contribute to the development of caries, type 2 diabetes and atherosclerosis [57]. A survey conducted by CBOS [59] revealed that 55% of Poles limit their sugar and sweets consumption. This result is similar to the results obtained in this research by people with proper eating habits (62.4%) and those non-food-focused (47.2%). Recommendations on limiting the consumption of meat, animal fats as well as sugar and sweets have been included among the principles of healthy nutrition developed by the Institute of Food and Nutrition (IŻŻ) experts [57]. The literature emphasizes that the diet of an average Pole deviates from recommendations on proper nutrition in the area of excessive consumption of the abovementioned products [60]. Therefore, nutritional restrictions undertaken by people with orthorexia and a tendency towards orthorexia should be considered desirable and beneficial to health as it could be assumed that they were undertaken not only because of taste preferences, however, but above all because of greater nutritional awareness. At the same time, it should be underlined, however, that excessive concentration on healthy eating can lead to the elimination of entire food groups from the diet, which in turn may have negative health consequences [61]. The subject’s literature presents various case reports of orthorexia, e.g., a case of 28-year-old who initially eliminated fats from her diet and then limited the consumption of other products [2], a 30-year-old man who limited his diet to brown rice and fresh vegetables only [11] and a 33-year-old woman whose diet consisted only of fresh fruit, vegetables and eggs [39]. In all the cases cited, extreme nutritional restrictions resulted in serious health consequences requiring medical intervention. Moreover, it should be emphasized that the desire to eat healthy food is not a disorder in itself, but excessive concentration on the quality of consumed products and the method of preparing meals, combined with the existence of negative consequences of such consumption, may lead to ON.

The definition of ON emphasizes that the obsession with healthy eating results from excessive health concerns [1], which have not yet been studied but determined only on the basis of patients’ observations. The results of our own research did not confirm this theory. Although people with a tendency towards orthorexia and people with orthorectic disorders showed a higher level of care for their health, and the results obtained by them indicate a tendency to maintain a positive attitude, they nevertheless fall within the range of neutral attitudes, similarly to people who eat properly and those not food-focused. No significant differences between orthorectic disorders and caring for health among young people aged 18–35 are visible in the study by Plichta and Jeżewska-Zychowicz either [27]. The result obtained by the authors of this study was surprising; however, after careful analysis of the used tools, it may have a logical justification. The Health Concern Scale expresses fears related to the impact of diet and, more precisely, the excessive consumption of substances such as sugar or fat on health. Thus, since people with orthorexia significantly reduced their intake, they also minimized their adverse effects on health. Therefore, the result regarding people with orthorectic disorders within neutral health attitudes may be justified.

Additionally, current research conducted among various groups indicates that health concerns increase with age and are most visible among the elderly and the sick [62,63]. Neutral attitudes towards health in the studied group may additionally be associated with the young age of respondents and lack of complaints related to improper nutrition; nevertheless, they are consistent with the results obtained by other authors who also focused on young respondents [64,65].

## 5. Research Limitations

The first encountered limitation is the availability of literature pertaining to health care and eating behaviors in orthorectic disorders [16,27]. Additionally, the conducted research also has its limitations, such as the inability to consider the research group as representative as their majority of respondents were female. Moreover, the validity of the ORTHO-15 questionnaire used to determine orthorectic disorders was recently questioned due to, among others, variable internal consistency (Cronbach’s alpha from 0.14 to 0.82; Cronbach’s alpha mean 0.55), contradictory credibility and doubts about the cut-off point [65,66,67,68]. Nevertheless, as noted earlier, it was the only tool available at the time of the research. Finally, the study had a cross-sectional design; therefore, it was impossible to draw conclusions as to the direction of the impact of the studied variables.

## 6. Conclusions

Conducted research revealed that the prevalence of orthorectic disorders in the studied group of young people is a relatively rare phenomenon, although a large number of respondents have a tendency towards this disorder. In addition, people with orthorectic disorders and a tendency towards orthorexia present neutral (with a tendency to positive) attitudes towards caring for their health. The obtained results may be the basis for further research on ON symptoms and may be helpful in understanding the specificity of this disorder.

It would be desirable to repeat the current studies in the future on a more representative group, using more reliable tools for orthorectic disorder assessment to verify the obtained results.

In addition, the nutritional behaviors observed in the studied group show some irregularities, which indicates the need for preventive and educational activities among young people aimed at increasing awareness of the role of proper nutrition.

Certainly, the greatest challenge for future research is the correct assessment of ON, which is undoubtedly difficult, because many of the behaviors characteristic of this disorder fall within the categories of normality and sometimes may even be laudable. The problem with precise diagnosis of this disorder may also result from distinguishing, on the basis of obtained results, only two groups, i.e., people exhibiting ON behaviors and those not exhibiting features of the disorder. Therefore, one of the major areas of future research would be to create a reliable diagnostic tool and to finally distinguish between orthorexia and overdiagnosis.

## Figures and Tables

**Table 1:** Division of the research group into four categories based on the ORTO-6 questionnaire.

Categories	Sex						OR (Women/Men)
	Women		Men		All		
	*n*	%	*n*	%	*n*	%	
Orthorectic symptoms as dominant	14	4.2	3	2.1	17	3.6	2.00
Tendency towards orthorectic disorders	101	30.5	37	26.1	138	29.2	1.17
Proper eating habits	157	47.4	53	37.3	210	44.4	1.27
Low food interest	59	17.8	49	34.5	108	22.8	1.93 *
All	331	70	142	30	473	100	-

*n*—number; *—Signifies the odd ratio Men/Women.

**Table 2:** Restrictions in consuming specific food products among all of the respondents, based on the ORTO-6 questionnaire.

I Limit:	All		Orthorectic Symptoms as Dominant	
	*n*	%	Chi-2	*p*
Amount of food	318	67.2	8.255	0.041
Food containing large amounts of fats	284	60.0	56.331	0.000
Fats	273	57.7	33.465	0.000
Sugar, Sweets	309	65.3	35.311	0.000
Grain products	90	19.0	7.789	0.051
Fish	53	11.2	3.214	0.360
Meat, sausage	109	23.0	15.118	0.002
Raw vegetables	41	8.7	1.940	0.585
Raw fruits	19	4.0	5.428	0.143
Dairy products	101	21.4	3.680	0.298

% do not add up to 100, because respondents could choose more than one answer; *n*—number; Chi-2—test; *p*—statistical significance.

**Table 3:** Restrictions of specific products in specified groups based on the ORTO-6 questionnaire.

I Limit:	Orthorectic Symptoms as Dominant		Tendency towards Orthorexia		Healthy Eating		Low Food Interest	
	*n*	%	*n*	%	*n*	%	*n*	%
Amount of food	12	70.6	103	74.6	141	67.1	62	57.4
Products high in fats	15	88.2	104	75.4	131	62.4	34	31.5
Fats	14	82.4	94	68.1	127	60.5	38	35.2
Sugar, Sweets	14	82.4	113	81.9	131	62.4	51	47.2
Grain products	3	17.6	37	26.8	34	16.2	16	14.8
Fish	0	0.0	13	9.4	26	12.4	14	13.0
Meat, sausages	7	41.2	44	31.9	43	20.5	15	13.9
Raw vegetables	0	0.0	11	8.0	20	9.5	10	9.3
Raw fruit	0	0.0	6	4.3	5	2.4	8	7.4
Dairy products	5	29.4	36	26.1	40	19.0	20	18.5

% do not add up to 100, because respondents could choose more than one answer; *n*—number.

**Table 4:** Caring for health among all respondents.

**Health Concern (HCS)**	**ORTO 6**								**ANOVA** **F, *p*, R.I. (Tukey Test)**
	**Orthorectic Symptoms as Dominant**		**Tendency towards Orthorexia**		**Healthy** **Eating**		**Low Food** **Focus**		
	**M**	**SD**	**M**	**SD**	**M**	**SD**	**M**	**SD**	
	47.65	12.23	47.89	11.69	44.01	11.73	43.93	12.34	3.248 0.022 2/4

M—average; SD—standard deviation; *p*—statistical significance.

## Data Availability

The data presented in this study are available from Natalia Kaźmierczak-Wojtaś.
